# Strengths and Weaknesses of Recently Engineered Red Fluorescent Proteins Evaluated in Live Cells Using Fluorescence Correlation Spectroscopy

**DOI:** 10.3390/ijms141020340

**Published:** 2013-10-14

**Authors:** Amanda P. Siegel, Michelle A. Baird, Michael W. Davidson, Richard N. Day

**Affiliations:** 1 Department of Cellular and Integrative Physiology, Indiana University School of Medicine, 635 Barnhill Dr., MS 333, Indianapolis, IN 46202, USA; E-Mail: apsiegel@iupui.edu; 2 National High Magnetic Field Laboratory and Department of Biological Science, 1800 E. Paul Dirac Dr., Florida State University, Tallahassee, FL 32310, USA; E-Mails: baird@magnet.fsu.edu (M.A.B.); davidson@magnet.fsu.edu (M.W.D.)

**Keywords:** fluorescent protein, fluorescent correlation spectroscopy (FCS), diffusion, intrinsic brightness, flickering

## Abstract

The scientific community is still looking for a bright, stable red fluorescent protein (FP) as functional as the current best derivatives of green fluorescent protein (GFP). The red FPs exploit the reduced background of cells imaged in the red region of the visible spectrum, but photophysical short comings have limited their use for some spectroscopic approaches. Introduced nearly a decade ago, mCherry remains the most often used red FP for fluorescence correlation spectroscopy (FCS) and other single molecule techniques, despite the advent of many newer red FPs. All red FPs suffer from complex photophysics involving reversible conversions to a dark state (flickering), a property that results in fairly low red FP quantum yields and potential interference with spectroscopic analyses including FCS. The current report describes assays developed to determine the best working conditions for, and to uncover the shortcoming of, four recently engineered red FPs for use in FCS and other diffusion and spectroscopic studies. All five red FPs assayed had potential shortcomings leading to the conclusion that the current best red FP for FCS is still mCherry. The assays developed here aim to enable the rapid evaluation of new red FPs and their smooth adaptation to live cell spectroscopic microscopy and nanoscopy.

## 1. Introduction

Fluorescent proteins (FPs) are valuable tools for analyzing cellular processes in living cells. The cloning of FPs first from *Aequoria victoria* and then from diverse marine organisms has led to an expanded palette of colors for multicolor imaging [1]. Red FPs take advantage of the lower autofluorescent background and reduced phototoxicity associated with longer-wavelength excitation. The first of the commercially available red FPs was mined from the sea anemone *Discosoma striata* and is commonly known as DsRed [2]. mCherry, derived by directed evolution of DsRed and first introduced nearly a decade ago [3], has been the most widely used red FP for FCS and other single molecule applications. While newer red FPs displaying increased brightness, photostability, monomeric quality, and speed of maturation have been engineered from DsRed and other anthozoa, most have not yet been evaluated for FCS or other single molecule applications. The goal of the current study is to develop a methodology that enables a careful scrutiny of newly developed red FPs for spectroscopic approaches by utilizing criteria important for these applications, and to determine their ideal conditions for use in live cell FCS.

All live cell assays require FPs that are nontoxic, do not interfere in cellular processes, and are truly monomeric [4]. However, there are additional or modified criteria that must be considered when selecting a red FP for single molecule applications. For example, low expression levels are a requirement for FCS, and ideal concentrations are in the nanomolar range. This is because the sensitivity becomes limited by increasing concentrations within the confocal volume. In the current study we evaluated red FPs using four criteria: photostability for FCS data collection; rate of diffusion of the FPs alone or when fused to other proteins and expressed in living cells; brightness as determined in live cells; and flickering, a process whereby red FPs undergo dark state conversions that can significantly affect FCS and other spectroscopic data collection [5]. The molecular brightness of the FPs in living cells was also used to assess their tendency to self-associate.

All red FPs consist of a central chromophore containing a phenol ring and an imidazolinone ring linked by a conjugated carbon, with additional conjugated bonds extending beyond the imidazolinone [6,7]. This chromophore is surrounded and stabilized by a structured β barrel. The main differences among red FPs are the varying amino acid residues at specific locations where the chromophore interacts with the β barrel. The red FPs used in this study include FusionRed [8], mRuby2 [9,10], mApple [11], and TagRFP-T [11,12]. mApple, like mCherry, was engineered from mRFP1, a derivative of DsRed. mRuby2, FusionRed and TagRFP-T were developed initially from two proteins derived from the sea anemone *Entacmaea quadricolor* [6,7]. mRuby2, developed from eqFP611, is over 2.5 times as bright as mCherry. TagRFP-T, from eqFP578, has a published brightness 3 times that of mCherry [13], while FusionRed (also from eqFP578) is equally bright as mCherry and has other advantages including low toxicity [8]. Here, we evaluated the photostability, flickering, molecular brightness, and diffusion characteristics of these red FPs using the purified FPs and the FPs expressed in mouse GHFT1 cells. To further analyze whether these red FPs can accurately report diffusion, they were fused to the leucine zipper (BZip) domain of the rat CCAAT/enhancer-binding protein alpha (C/EBPα) [14], as described below.

## 2. Results and Discussion

### 2.1. Autocorrelation Curves of Dyes and Purified Proteins in Solution

#### 2.1.1. Autocorrelation Curves at a Range of Laser Powers

FCS analyzes fluctuations in intensity within a small confocal volume measured at rapid (μs) intervals. The fluctuations in fluorescence intensity over time are fit to an autocorrelation curve that provides information about molecular diffusion. The simplest FCS curve-fitting uses a model that assumes that all intensity fluctuations within a confocal volume arise due to molecular movement, and is shown here as Equation (1)

(1) $$G(\tau )=\frac{1}{N}{\left[1+\frac{\tau }{\tau_{CHAR}}\right]}^{-1}{\left[1+\frac{1}{Q^{2}}\frac{\tau }{\tau_{CHAR}}\right]}^{-0.5}$$

where *G*(τ) represents the normalized variation in intensity from average intensity measured at *t* + τ for all values of *t*, *N* represents the number of molecules in the confocal volume, *Q* represents a shape-fitting parameter and τ*_CHAR_* is the characteristic diffusion time for the species, assuming it is freely diffusing.

**Figure 1:**
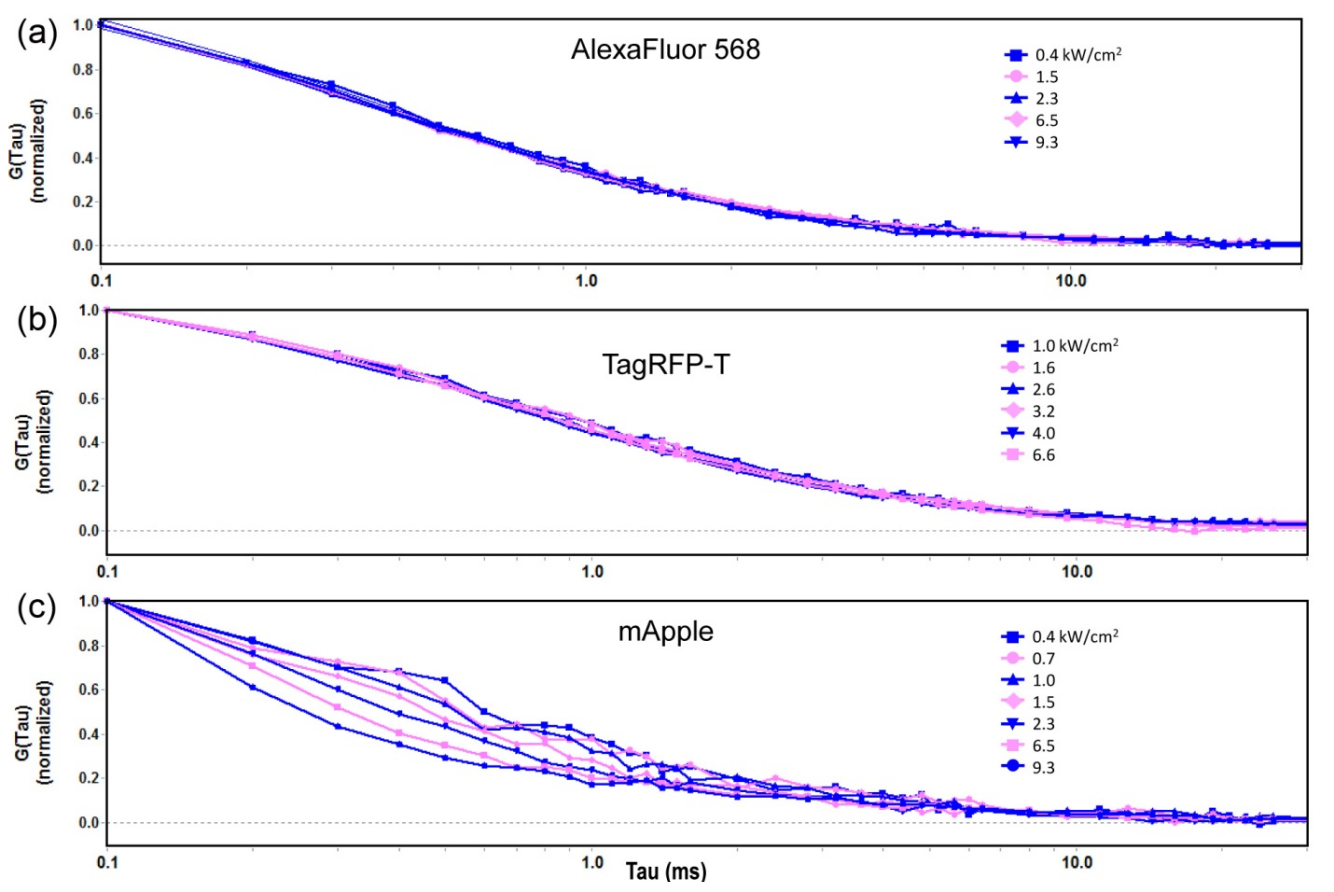
(**a**–**c**) Normalized autocorrelation curves for AlexaFluor 568, TagRFP-T and mApple in PBS over the indicated range of laser power densities. Since the rate of molecular diffusion is independent of laser intensity, the changes in the autocorrelation curves for mApple indicate other molecular activities, such as flickering, are interfering with the FCS measurement.

However, fluctuations in the emission intensity of a fluorophore occur for reasons other than diffusion. Intensity fluctuations that occur on different time scales than molecular movement, such as triplet conversions or fluorophore bleaching, can be accounted for by varying the rate of acquisition or total acquisition time. Alternatively, more sophisticated FCS models can be utilized to determine the triplet contribution. Intensity fluctuations that occur on the same time scale as τ*_CHAR_*, however, interfere significantly with FCS analysis. One way to determine whether FCS measurements accurately reflect molecular diffusion is to test the model system using different laser excitation powers. Non-diffusion related intensity fluctuations, whether due to triplet formation, bleaching, or other molecular activities, will vary with laser intensity, whereas the rate of diffusion of the fluorophores will not. Figure 1a shows normalized FCS autocorrelation data for AlexaFluor568 at a range of laser intensities measured at the objective. The results demonstrate that this molecule does not undergo significant fluctuations with changes in laser intensity. The fitting data acquired over a 20-fold range of power densities (0.16 to 3.5 kW/cm^2^) using Equation (1) show characteristic diffusion times (τ_CHAR_) of 378 ± 14 μs. These results also demonstrate that the one-component fit described by Equation (1) is adequate to model a freely diffusing dye at these laser intensities.

The model was then tested for the measurements of the diffusion characteristics of the red FPs. Other studies have reported that some red FPs undergo a sub-millisecond fluctuation between a fluorescent state and a dark state, which is called flickering [5,13,15,16]. Figure 1b shows the autocorrelation curves for TagRFP-T in solution at a range of laser intensities from 0.22 to 1.48 kW/cm^2^. The results indicate that this red FP does not undergo dramatic fluctuations (flickering) over this range in laser intensity. In stark contrast, mApple shows a clear variation in the shape of the autocorrelation curves with changing excitation intensity. Figure 1c shows autocorrelation curves for mApple at a range of laser intensities from 0.16 to 3.5 kW/cm^2^ demonstrating that as the laser power increases, there are more fluctuations. These are especially evident in the range from τ = 0.2–2.0 ms, where the fluctuations interfere with the determination of τ_CHAR_, which is the basis for calculating *D* (described in the Methods section). The source of red FP flickering is likely *cis-trans* isomerization of the bond between the phenol and the imidazoline rings, or rotations of the rings themselves in the red FP chromophore contained within the β barrel [5,17]. Different amino acid residues on the β barrel that interact with the chromophore significantly affect the flickering [13].

#### 2.1.2. The Effect of Flickering on Red FP Autocorrelation Curves

We hypothesized that if flickering is a function of the excitation intensity, then it should be possible to control these fluctuations in intensity by limiting the excitation power. Therefore, we first determined the rate of diffusion of the red FPs at the lowest excitation intensities capable of providing adequate signal-to-noise ratios, and analyzed the autocorrelation functions using Equation (1). There are two reasons why the one-component FCS equation was used for this analysis. First, the studies at varying laser intensities showed that this equation was capable of determining τ_CHAR_ and therefore *D* with reasonable accuracy. Second, the use of a triplet-fitting algorithm could mistake flickering fluctuations for triplet fluctuations and would therefore be a less suitable method for analyzing the FCS data for these species. When this method was applied to the FPs in this study, four of the five red FPs gave the same value for *D*, within experimental error. In contrast, the *D* for mApple was approximately 25% higher than that for other FPs despite having the same hydrodynamic size. This shows that the flickering of mApple was interfering with analysis of the autocorrelation curve even at the lowest excitation intensities. This finding is likely related to the complex photo-switching behavior of mApple reported previously [11]. There, photo-switching (flickering) was manifested as a rapid decrease in total fluorescence that was reversible. The study by Shaner [11] is notable because unlike most reported studies of red FP flickering, it utilizes excitation intensities sufficiently low that the flickering can be investigated without the need to account for the effect of photolysis. The current study also indicates reversible photo-switching, determined by the evidence of the autocorrelation curves in Figure 1.

**Figure 2:**
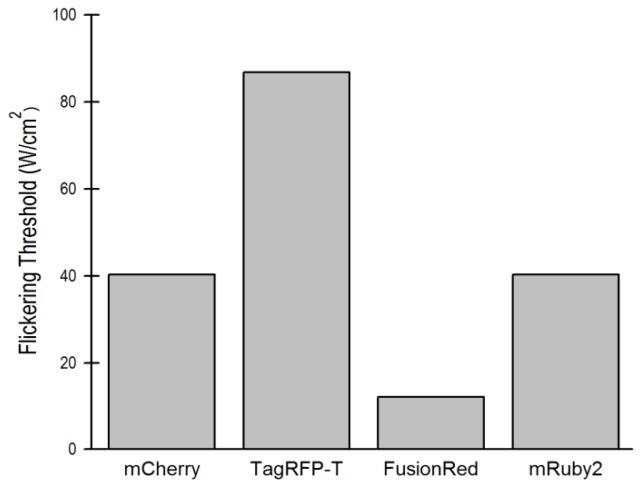
The flickering threshold for four of the five red FPs tested. mApple was excluded because it flickers at all laser powers tested.

We next varied excitation intensities for the other red FPs to further investigate their flickering characteristics. For each of the four remaining red FPs, we were able to find a threshold below which change in excitation intensity did not change *D*. When the excitation intensity was increased above this threshold, which we define as the flickering threshold, an artificially high *D* was measured. Representative autocorrelation curves and a table showing values for *D* at different excitation intensities for the four red FPs at ranges of laser intensity are in the Supplementary Material. These data were used to determine the flickering thresholds for mCherry, TagRFP-T, FusionRed and mRuby2 (Figure 2 and Table 1). FusionRed was most susceptible to flickering (variations in *D* noted above 0.11 kW/cm^2^), followed by mRuby2 and mCherry, while TagRFP-T displayed very little flickering. Table 1 gives the *D* values for the purified proteins determined by averaging all the readings below the flickering thresholds, except for mApple, where the *D* value was determined at 0.075 kW/cm^2^. There was little evidence of photobleaching while acquiring these measurements. Photobleaching is a separate characteristic of the FPs and is not necessarily correlated to flickering [5]. This distinguishes these results from other studies performed at higher illuminations where flickering and photolysis (and triplet effects) occur simultaneously, and are separated using different analytical techniques [5,13,15].

### 2.2. Optimization of Excitation Intensity in Live Cells

FCS was then performed in live cells expressing the different red FPs to determine the optimal excitation intensity for each protein. The cells were selected for analysis by epi-fluorescence imaging (using a Texas Red excitation filter on a mercury arc lamp attenuated with a 75% neutral density filter). The autocorrelation curves were then acquired over a range of laser powers. The maximum counts per second (cps) collected for the FCS measurements were limited to minimize detector afterpulsing, which can introduce artifacts into an autocorrelation curve [18]. Because of the molecular brightness of TagRFP-T, this limited the optimal excitation power that could be used. The flickering thresholds determined for the purified proteins (described above) were found to be applicable to the measurements in the live cells: for red FPs other than mApple, the optimal excitation intensity was found below the flickering threshold (Figure 2). Values for optimal excitation intensity are shown in Table 1. The important methodological point is to identify the laser powers below the flickering threshold, below excitation intensities that induce bleaching, with total count rates below those that induce afterpulsing according to the manufacturer’s recommendations, yet high enough that the background signal does not contribute significantly to the autocorrelation curve.

### 2.3. FP Diffusion Studies

**Figure 3:**
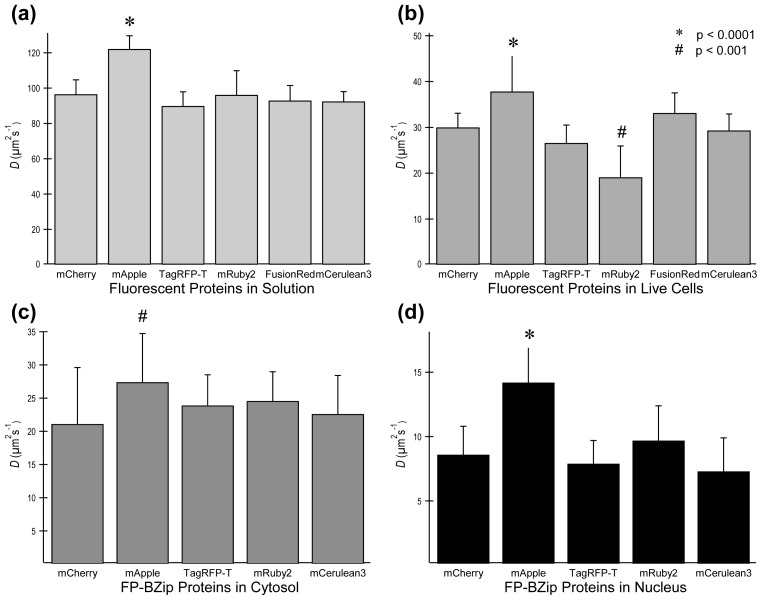
Diffusion coefficient (*D*) determined by FCS at optimal excitation intensities (see Table 1). (**a**) *D*s for purified FPs in solution are all statistically the same, except for mApple, which has no flickering threshold and shows an apparent *D* 30% > than *D* for other FPs; (**b**) *D* for FPs determined in cytosol at optimal excitation intensities. The apparent *D* for mApple-BZip is significantly (*) higher than the other FP-fusion proteins. *D* for mRuby2 is significantly (#) lower than the other FPs; (**c**) *D* for FP-BZip fusion proteins determined in the cytosol. The apparent *D* for mApple-BZip is significantly (#) higher than the other FP-BZip fusion proteins; (**d**) *D* for FP-BZip fusion proteins in the nucleus (away from heterochromatin) is much slower than in the cytosol due to BZip interactions with DNA. Apparent *D* for mApple=BZip is significantly (*) higher. Significance determined by comparing each red FP against values found for mCerulean3 in solution and in live cells for multiple *T* tests using the Holm-Sidak method (* *p* < 0.001, # *p* < 0.0001).

Table 1 and Figure 3 show values for *D* found in solution and in live cells for the five different red FPs. For comparison purposes, *D* was also determined for mCerulean3, a cyan FP that is quite photostable and is not excited by the 561 nm laser line [19]. Because its maximum emission is below 520 nm, and these red FPs do not emit below 520 nm when excited with a 561 nm laser, it is possible to select appropriate filters to use mCerulean3 sequentially with red FPs with no overlap of fluorescent signal, thus enabling dual color FCS studies [14]. Each of the purified red FPs, with the exception of mApple, had the same value for *D* as mCerulean3 (Table 1 and Figure 3a). In contrast, measurements of diffusion of the red FPs expressed in the cytoplasm of living cells demonstrated differences in the diffusion rate both slower and faster than that measured for mCerulean3 (Figure 3b) Data acquired in the nucleus gave similar diffusion values to data acquired in the cytoplasm (data not shown). mRuby2 diffused at a much slower rate in the cytoplasm of live cells (19.1 ± 3.6 μm^2^/s), whereas mApple gave a much larger *D* (37.8 ± 8.5 μm^2^/s). The larger *D* for mApple is likely due to the same flickering processes that caused the incorrect determination of *D* for mApple in solution. The much lower value of *D* for mRuby2 requires further analysis.

The ratio of the diffusion coefficient for the purified FPs in solution (*D_pur_*) and the diffusion coefficient in the cytoplasm of cells (*D_cytoplasm_*) is a useful metric that should accurately reflect the change in viscosity and temperature between the two media. The Stokes-Einstein equation states that *D* = *κT/6πηr*, where *κ* is the Boltzmann constant, *T* is the absolute temperature, *r* is the hydrodynamic radius of the diffusing particle, and *η* is the viscosity of the solution. This equation predicts that for any diffusing particle, *D_cytoplasm_:D_pur_* should be a constant provided that there is no interaction of the FP with cellular components. The results show that the measured ratios *D_cytoplasm_:D_pur_* for mApple, TagRFP-T, mCherry and mCerulean3 were statistically the same (mCerulean3, 32% ± 6%; mCherry, 31% ± 4%; mApple, 31% ± 7%; TagRFP-T, 30% ± 5%). In contrast, the measured ratio *D_cytoplasm_:D_pur_* for mRuby2 was much smaller (20% ± 4%). Both by measured *D* in live cells, and by comparing the change in *D* between solution and in live cells, it is evident that mRuby2 diffuses more slowly than other FPs in the cytoplasm of living cells. Similar results were also obtained with mRuby (not shown), the precursor to mRuby2. This is unfortunate because mRuby2 has been extensively engineered for brightness, and is among the brightest of current monomeric red FPs [9]. The decrease in the rate of diffusion could be caused by mRuby2 aggregation or interactions with other proteins in the cytosol, and is considered more fully in Section 2.4.

**Figure 4:**
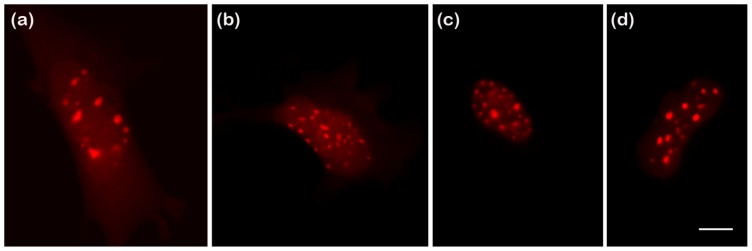
Epifluorescent images of GHFT1 cells expressing FP-BZip fusion proteins. All four red FP-BZip fusion proteins had the expected localization to regions of heterochromatin. FCS data for Figure 3c,d were obtained in the cytosol and in the nucleus away from the heterochromatin bundles. (**a**) mCherry-BZip; (**b**) mApple-BZip; (**c**) TagRFP-T-BZip; and (**d**) mRuby2-BZip. Scale bar = 10 μm.

Next, the ability of the different red FPs to report *D* values for fusion proteins was probed using the previously characterized BZip transcription factors [14], and the combined results are shown in Figure 3c,d and Table 1. BZip transcription factors homo or heterodimerize by forming a parallel coiled coil (a “leucine zipper”), and bind to specific DNA elements using a region rich in basic amino acids [20]. The BZip domain diffuses at three different rates in live cells: in the cytosol its diffusion is essentially unhindered; in the nucleus away from heterochromatin it diffuses more slowly than in the cytoplasm; and in regions of heterochromatin, it binds as an obligate dimer for a significant time and diffusion is very slow. For the current studies, FCS diffusion data were collected in the cytoplasm and in the nucleus away from heterochromatin to determine how well the red FPs could report diffusion of the fusion proteins. The results for the red FP-BZip fusion proteins were compared with the results from mCerulean3-BZip fusion protein, which was used earlier to characterize the mobility of the BZip proteins [14]. Due to its low intrinsic brightness and low flickering threshold, FusionRed was not utilized in these diffusion tests. Figure 4 demonstrates that each of the four red FPs fused to the BZip domain showed the appropriate localization to heterochromatin that is expected for BZip fusion proteins [14]. Because the diffusion of the BZip fusion protein in the cytosol is unhindered by DNA binding events, the expected *D* for FP-BZip proteins is governed only by hydrodynamic radius. Due to the small size of the BZip domain (13 kDa) it is expected that *D* for FP-BZip will only be slightly slower than *D* of the FP alone. In contrast, BZip diffusion in the nucleus will be slower than in the cytosol due to transient interactions with DNA and possibly nuclear crowding [21]. The results in Figure 3 show that mApple-BZip had significantly higher *D* than the rest of the FP-BZips both in the cytosol and in the nucleus. In the cytosol, each of the other red FP-BZip fusion proteins gave the same rate of diffusion as mCerulean3-BZip, indicating all were capable of reporting a correct *D* for the fusion protein. In the nucleus away from heterochromatin, each of the red FP-BZip proteins (other than mApple) diffused at about the same rate as mCerulean3-BZip.

### 2.4. Brightness Analysis in Live Cells

**Figure 5:**
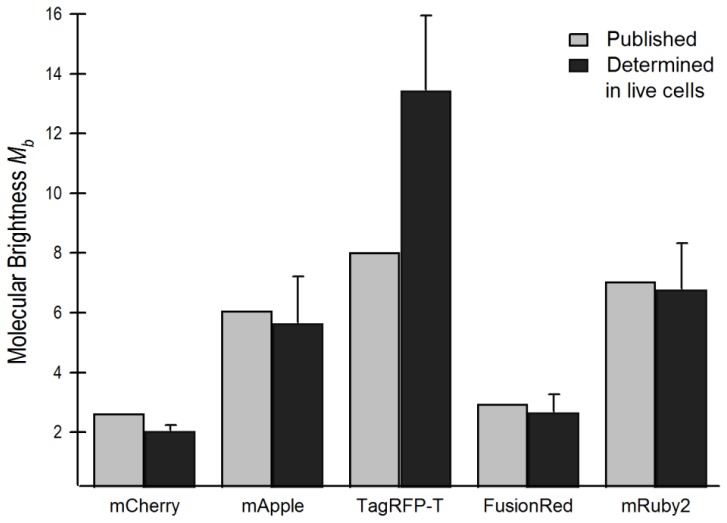
Experimentally determined molecular brightness, *M_b_*, of red FPs in live cells compared with published intrinsic brightness values normalized by a scalar factor (see text). *M_b_* values for all FPs except mApple were found using one-component autocorrelation curves to determine concentration, (see text for discussion of determination of *M_b_* for mApple).

We next used the FCS autocorrelation curves to estimate the average *N* within the confocal volume, and thus used them to determine molecular brightness (*M_b_*), which is defined as follows:

(2) $$M_{b}=\frac{CPS}{N*P_{o}}$$

where CPS is the average total intensity and *P_o_* is the power at the objective. For example, for one measurement of mRuby2, CPS was 11,084, *N* was 1.4, and *P_o_* was 0.0012 mW. *M_b_* determined by FCS should be directly correlated, using a scale factor (described below), to the intrinsic brightness of the FPs, which is found by multiplying quantum yield (QY) and extinction coefficient (EC). Here, the values for *M_b_* are compared with the published values of intrinsic brightness based on QY and EC (Figure 5 and Table 1). (Because mApple gave an incorrect *D* with a one-component diffusion model, the data were fit using a diffusion model in which *N* was determined by including a flickering component and setting *D* for the main component equal to *D_mCherry_* (see Section 3.2 and Equation (4)). The scale factor relating *M_b_* to intrinsic brightness was set by equating *M_b_* of AlexaFluor 568 in solution (determined by FCS) with its published intrinsic brightness (Invitrogen, division of Life Technologies). There are two published values for the intrinsic brightness of TagRFP-T that differ significantly in both EC and QY [11,13]. Figure 5 and Table 1 utilize the larger value for brightness reported by Dean *et al*. [13]. The *M_b_* and published values correspond reasonably well for four of the five FPs. The fifth FP, TagRFP-T was found to be far brighter per molecule in live cells than either of its published brightness values, and was in fact brighter, per molecule, than AlexaFluor 568 dye in solution.

It is also possible to determine brightness of the BZip-FPs by the same method. The BZip proteins form obligate dimers when bound to DNA, and our earlier FRET analysis showed conclusively that these BZip proteins do interact when bound to heterochromatin [14,22]. However, when diffusing with the cytosol, the BZip proteins are unable to form leucine zippers and are expected to be monomeric. To test this, *M_b_* for the mCherry-, mRuby2- and TagRFP-T-BZip proteins in the cytosol were determined using Equation (2). The results are included in Table 1, and indicate that mRuby2 and mCherry display nearly equivalent brightness values when expressed alone and as FP-BZip fusion proteins. In contrast, TagRFP-T is only 73% as bright when expressed as a BZip fusion protein as when expressed alone. The significance of this observation is discussed in Section 2.4.1. The mCherry and mRuby2 results verify the conclusion that BZip proteins do not form dimers while in the cytosol.

#### 2.4.1. Using Brightness to Determine Oligomeric State

The fact that mRuby2 diffuses at the same rate as the other red FPs in solution, but moves at a slower rate in live cells, might be due to homo-aggregation or interactions with cellular proteins. Brightness analysis can identify molecular aggregation of FPs. The *M_b_* value determined for mRuby2 in live cells is similar to its published intrinsic brightness. This is evidence that mRuby2 is monomeric in live cells. In further support, the *M_b_* value for mRuby2 was equal to the *M_b_* value for the fusion protein mRuby2-BZip. Based on the brightness studies, it seems likely that mRuby2 does not homo-aggregate. Therefore, the best explanation for the mRuby2 rate of diffusion is that it appears to be undergoing transient interactions with cellular proteins that slow it down. This could diminish its utility as a fluorophore in binding or diffusion studies.

**Figure 6:**
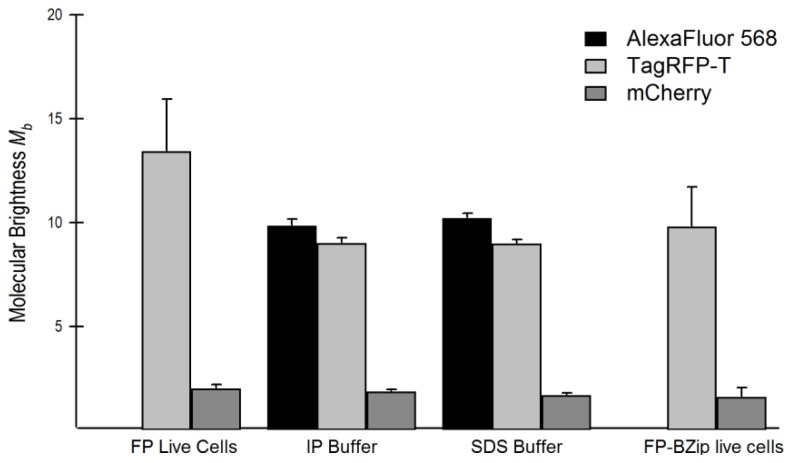
Molecular brightness (*M_b_*) of different fluorophores determined by FCS in different conditions. AlexaFluor 568 and mCherry brightness are unchanged in all conditions. TagRFP-T is ~30% brighter when measured in live cells compared with brightness of lysates in IP buffer, SDS buffer, or when expressed in live cells as TagRFP-T-BZip.

In contrast to mRuby2, the *M_b_* value determined for TagRFP-T expressed alone in live cells is much larger than its published intrinsic brightness, and is larger than the *M_b_* value determined for the fusion protein TagRFP-T-BZip. One explanation is some fraction of TagRFP-T may dimerize when expressed in living cells, which would increase its average brightness. An FP isolated from the same coral as TagRFP-T, eqFP611, was shown to dimerize, but could be disassociated with SDS treatment [23]. To test whether TagRFP-T forms dimers or higher order aggregates in cells, lysates of GHFT1 cells containing TagRFP-T were prepared and diluted in an immunoprecipitation (IP) buffer containing the detergent NP-40, and the lysates were analyzed by FCS. Next, SDS was added to the IP buffer to 0.1% *w*/*v* and the brightness of TagRFP-T was again determined. For comparison purposes, lysates of cells expressing mCherry and AlexaFluor 568 prepared in the same buffers were similarly analyzed. The results are shown in Figure 6, which also includes *M_b_* of TagRFP-T and mCherry expressed alone and as FP-BZip fusion proteins. These results show that TagRFP-T expressed alone in live cells was about 30% brighter than TagRFP-T in lysates with detergent or when conjugated to BZip. There was no similar diminution in brightness shown by mCherry or AlexaFluor568 under similar conditions. These results strongly suggest that a fraction of TagRFP-T self-associates in live cells, but that this can be disrupted by the addition of detergent. Since the TagRFP-T-BZip fusion proteins were about as bright as the TagRFP-T lysates in buffer, the BZip protein may be acting to prevent the weak self-association of TagRFP-T. Curiously, the TagRFP-T lysates and TagRFP-T-BZip fusion proteins are still brighter than the published values for the intrinsic brightness of TagRFP-T [11,13].

Brightness analysis therefore strongly suggests that a fraction of TagRFP-T self-associates in live cells. Diffusion analysis, by contrast, cannot easily detect a fraction of molecules diffusing as dimers. Because diffusion varies as the hydrodynamic radius, and generally speaking the hydrodynamic radius varies as the cube root of molecular weight (monomer is 27 kDa), if all the molecules were dimers (54 kDa) they would diffuse just 21% slower than monomers. If only a fraction self-associate, decrease in diffusion is correspondingly less. If approximately 30% of the TagRFP-T proteins are dimers, as suggested by the brightness analysis, this would cause the average *D* for the whole population of TagRFP-T to decrease only about 6%, which is close to experimental error. In summary, the brightness of TagRFP-T expressed in live cells compared with its brightness in other conditions strongly suggests a fraction of TagRFP-T self-associates in live cells, which is not evident from analysis of TagRFP-T diffusion alone. The literature lends some support for a finding that TagRFP-T may form dimers. Despite the initial reports that TagRFP and TagRFP-T were monomers [11,12], subsequent studies showed that TagRFP (which differs in one amino acid residue from TagRFP-T) had a mixed population of monomers and dimers in a semi-native polyacrylamide gel [24].

### 2.5. Relationship of Intrinsic Brightness to Flickering Threshold

**Figure 7:**
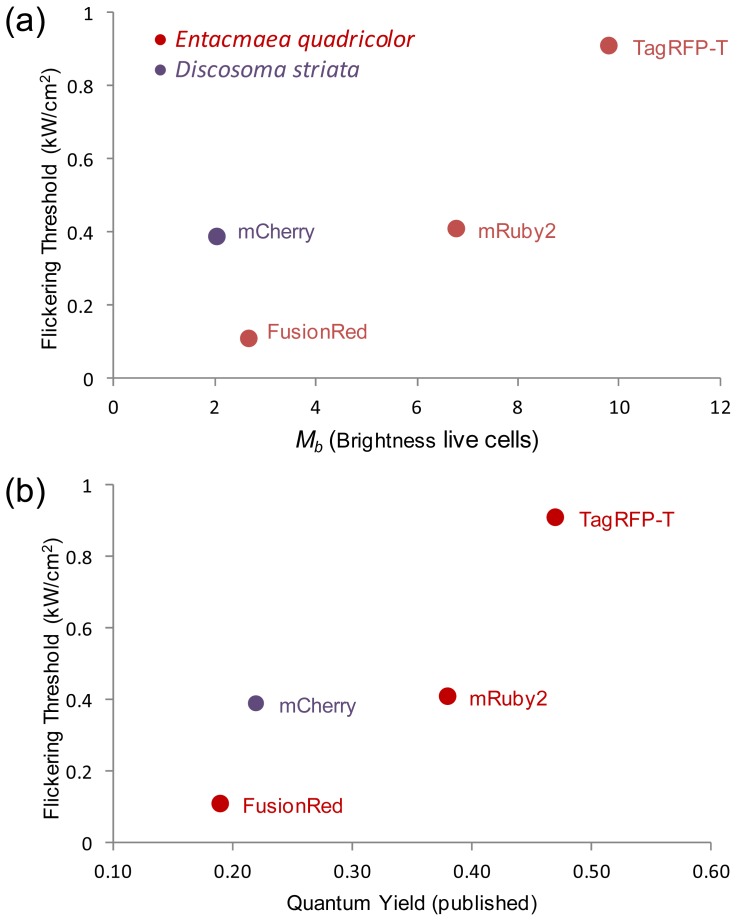
Comparison of photophysical parameters of red FPs from two marine organisms showing the correlation between flickering threshold and both *M_b_* (**a**); and quantum yield (**b**) for the red FPs derived from *Entacmea quadricolor*.

Determination of the flickering threshold was pursued *a priori* as a requirement for obtaining accurate FCS results from the red FPs, but it also uncovers an interesting relationship. There is a possible trend relating the flickering threshold and molecular brightness, and the flickering threshold and measured quantum yield, of the three red FPs from *Entacmaea quadricolor* (labeled in red in Figure 7) (here, the *M_b_* used for TagRFP-T is the brightness found in lysate with detergent). The flickering threshold can be considered the excitation intensity at which the chromophore starts to dissipate a significant fraction of its excited state by a nonradiative process, likely by *cis-trans* isomerization or rotation of the phenol or imidazolinone ring. The non-radiative processes are affected by the specific amino acid residues on the FP β barrel [13]. Thus, determination of changes in the flickering threshold may help quantify the effect of specific amino acid residues on chromophore stabilization within the β barrel. The mCherry flickering has been investigated by at least three different groups [5,13,17], analyzing different aspects of the phenomenon. In the current study, the characteristic property of interest is the ratio of flickering threshold to brightness. Unlike the three FPs from *E. quadricolor*, mCherry has a higher ratio of flickering threshold to brightness (that is, it is fairly resistant to flickering even though it is not very bright). This suggests that further optimization of mCherry to increase the flickering threshold may also increase brightness.

## 3. Experimental Section

### 3.1. Protein Expression

For expression in mouse GHFT1 cells, all sequences encoding the FPs were in the Clontech N1 vector. For fusion proteins mApple-BZip, mRuby2-BZip, TagRFP-T-BZip, mCherry-BZip, and mCerulean3-BZip, the indicated FPs were linked in reading frame to the rat C/EBPα BZip domain, starting at position 237 of the full length C/EBPα protein. For purification of the FPs, the coding sequences were inserted in the constitutive expression vector pNCS (Addgene), which encodes an *N*-terminal 6xHis tag and linker, and subsequently expressed and purified as described previously [25]. The purified 6xHis TagRFP-T was kindly provided by Dr. John Murray (University of Indiana, Bloomington, IN, USA).

### 3.2. Tissue Culture and Transfection

Mouse pituitary GHFT1 cells were maintained in monolayer culture and harvested at 80% confluence. For transfection by electroporation the cells were washed with PBS and briefly treated with trypsin, and then recovered by centrifugation in culture medium containing serum. The cell pellet was washed two times by centrifugation in Dulbecco’s calcium-magnesium free PBS and resuspended in Dulbecco’s calcium-magnesium free PBS with 0.1% glucose and 0.1 ng/mL BioBrene Plus (Applied Biosystems, Inc., Division of Life Technologies, Carlsbad, CA, USA) at a final concentration of approximately 1 × 10^7^ cells/mL. Next, 400 μL of cell suspension was transferred to each 0.2 cm gap electroporation curvette containing 0.5–2 μg of plasmid DNA(s), and after gentle mixing, the cuvettes were pulsed with 200 V at a capacitance of 1200 μF in a BTZ ECM 830 electroporator (Harvard Apparatus, Holliston, MA, USA), yielding pulse durations of about 10 ms. The cells were recovered from the cuvette and diluted in phenol red-free tissue culture medium containing serum and transferred to sterile 2 well chambered coverglasses (Lab-Tek II, Thermo Fisher Scientific, Waltham, MA, USA). Cells were imaged 20–28 h after transfection.

For analyzing diffusion of FPs in lysates, the transfected cells were placed on ice for 20 min, and then rinsed 2× with ice cold PBS. 200–400 μL of immunoprecipitation (IP) buffer (50 mM Tris, pH8, 150 mM NaCl, 1 mM EDTA, 10% glycerol, 1× Protease Inhibitors (Sigma-Aldrich, St. Louis, MO, USA), 43 μM NP-40 (Boston Bioproducts, Ashland, MA, USA) was added and cells were manually scraped and collected into microcentrifuge tubes. The lysates were frozen at −80 °C and thawed and spun at 13,200 RPM for 10 min at 4 °C, and the supernatant was collected. The supernatant was diluted with more IP buffer or IP buffer with sodium dodecyl sulfate (SDS) to a concentration of 0.1%. Lysates of non-transfected cells were analyzed: the background autofluorescence signal did not contribute significantly to the autocorrelation curves generated by the fluorescent proteins.

### 3.3. FCS Measurements and Image Acquisition

The FCS measurements were made as described previously [14]. In brief, the ISS Alba FastFLIM system (ISS Inc., Champagne, IL, USA) was coupled to an Olympus IX71 microscope with a 60 ×/1.2 NA water-immersion objective with a Pathology Devices stage-top environmental control system to maintain temperature at 36 °C and CO_2_ at 5%. For epifluorescent image acquisition, a Hg lamp with a 75% ND filter was used as the excitation source and images were acquired on a Hamamatsu Orca-R^2^ C10600 digital CCD camera using Metamorph software. For FCS measurements, the system was equipped with a dual dichroic (445/561) beamsplitter. The laser beams (448 nm for mCerulean3 measurements, 561 for red FPs were focused to a confocal spot with a small excitation geometry (typical *ω_0_* = 0.45 μm). The laser power was determined at the specimen plane with a digital laser power and energy meter (Thorlabs PM100D, Newton, NJ, USA). For experiments depending on the power density at the objective, laser power readings were taken at the start of the session, periodically during a session, and at the end of a session. Power density is determined by dividing the laser power (typically between 0.5–2 μW) by the confocal cross-section (π × ω_0_^2^). The fluorescence signals emitted from the focal spot are routed via the beam splitter to two band-pass emission filters (480/40 and 609/54), and are then passed to two identical avalanche photodiodes with adjustable pinholes set at 50 μm. Emission events per time were collected at 10 kHz (in solution) or 2 kHz (in live cells) and used to generate autocorrelation and cross-correlation curves from the intensity traces (*I*(t)) and the fluctuations in the intensity (δ*I*(t) = *I*(t) − 〈*I*(t)〉). The curves were best fits for the general correlation function:

(3) $$G_{xy}(\tau )=\frac{\langle \delta I_{x}(t)\delta I_{y}(t+\tau )\rangle }{\langle I_{x}\rangle \langle I_{y}\rangle }$$

when *x* = *y*, this returns the autocorrelation function, and when *x* ≠ *y* this measures the cross-correlation of signals between two different channels. Standard dyes were used to determine the geometry of the confocal spot from the parameters τ*_CHAR_* and *Q* via the Equation (1). The beam waist (ω*_0_*) was calculated using the relationship τ*_CHAR_* = ω*_0_^2^/*4*D* and beam length (*z_0_*) from the relationship *Q* = *z_0_*/ω*_0_*. N is the concentration corrected by a geometric factor that is commonly used to take into account the uncertainty of the actual size of the confocal volume. The calibration standards are 2 nM Coumarin 6 (in HPLC grade EtOH (448 nm laser line, *D* = 396 μm^2^s^−1^) and 10 nM AlexaFluor 568 in PBS (561 laser line, *D* = 363 μm^2^s^−1^). These parameters are not dependent on laser power at the power levels utilized, but do vary from session to session, and so were calculated at each session. For the 561 line, typical values for ω*_0_* and *Q* are 0.45 μm and 6.0, and for the 448 line, ω*_0_* is generally smaller, around 0.39 μm. The purified FPs were diluted in PBS with 0.2% BSA to approximately 1 nM concentration.

The following equation enables the fitting of an autocorrelation curve including a triplet or flickering term:

(4) $$G_{triplet}(t)=\frac{1}{N}\left[1+A_{triplet}e^{\left(-\tau /\tau_{triplet}\right)}\right]\;{\left[1+\frac{\tau }{\tau_{CHAR}}\right]}^{-1}{\left[1+\frac{1}{Q^{2}}\frac{\tau }{\tau_{CHAR}}\right]}^{-0.5}$$

where *A_triplet_* = *F_triplet_*/(1 − *F_triplet_*), and *F* is the fraction of molecules residing in the dark state at any time [5]. Triplet effects involve excited photons relaxing to a forbidden triplet state. The flickering exhibited by red FPs is likely not triplet effects, but the same fitting equation can be used [5]. Determination of the maximum laser power density that can be utilized without flickering in the autocorrelation curve was carried out by determining *D* at increasing excitation intensities until the rate of diffusion fit by the autocorrelation curve became statistically significantly higher with the increase in laser power. Care was taken between readings (by using sufficient total volume of media and including waiting a short time between taking measurements) to ensure that the medium containing the sample was not heated by the laser, as that will also lead to higher reported *D.*

The diffusion of a molecule is related to its size by the Stokes-Einstein equation (*D = kT*/*6πηr*). The hydrodynamic radius can be estimated by

(5) $$r=\sqrt[{3}]{\frac{3m/N_{A}}{4\pi \rho }}$$

where *m* is the molecular weight, *N_A_* is Avogadro’s number, and *ρ* is the density of molecules. The hydrodynamic radius is not the exact radius of a molecule, but rather the perceived size of the molecule as it diffuses through a solution, assuming it obeys the Stokes-Einstein equation. Estimates of the density of proteins vary depending on amino acid composition, but proteins of very similar molecular shape and molecular weight, such as mCherry, mApple and mRuby2, would be expected to have very similar radii and similar *D* in solution.

The relative viscosities of two media can be determined by finding the ratio of *D* for the same molecule in the different media, for example η*_livecell_*/η*_soln_* = *D_soln_*/*D_livecell_*. Equation (5) also demonstrates that if the molecular weight of a protein is doubled, as in the formation of a dimer, *D* should decrease by a factor of 2^1/3^, or 1.26. Dimer formation, however, causes a change in configuration that may cause the change in hydrodynamic radius to be smaller than calculated using Equation (5), so this value is no more than a rough approximation and the decrease in diffusion might be even less than a factor of 1.26. The expected value for the ratio *D_soln_/D_livecell_* if a protein is a monomer in solution and a dimer in live cells is therefore η*_livecell_/*η*_soln_* = 1.26, but it could be less.

## 4. Conclusions

Red FPs are becoming increasingly important for techniques beyond multi-color analyses of protein subcellular localization. However, many of the quantitative spectroscopic techniques such as FCS require optimal biophysical behavior. Here, we combined several assays to evaluate the strengths and weaknesses of the current brightest red FPs for FCS and other single molecule and spectroscopic applications. These tests included determination of the flickering threshold of the red FPs, which varied significantly from mApple, which flickered at even the lowest excitation intensities, to TagRFP-T, which was most resistant to flickering. The flickering threshold is a good proxy for determining the energy barrier a chromophore must overcome to undergo non-radiative (flickering) processes. With the exception of mApple, flickering thresholds correlated well with molecular brightness and quantum yield of the FPs. In addition to determining flickering thresholds and optimal power density for FCS data collection, the comparison of FCS measurements in solution and inside living cells showed how well each of the red FPs performed, while also highlighting some of their shortcomings. For example, mApple underwent flickering at all excitation intensities, making it wholly unsuitable for FCS studies. FusionRed was shown to have a low flickering threshold, which combined with its relatively poor intrinsic brightness makes it a poor candidate for FCS studies as well. mRuby2 was very bright and had an acceptable flickering threshold, but when expressed in cells showed a rate of diffusion significantly slower than all of the other FPs. This indicates mRuby2 would be a poor choice of FP to study protein diffusion. Finally, the brightness analyses strongly suggest that TagRFP-T is not entirely monomeric when expressed in live cells, although TagRFP-T did appear to diffuse as a monomer when fused to the BZip protein, and others have utilized it to study spatiotemporal colocalization [26]. Based on the shortcomings determined here, we believe the current best red FP for FCS studies may still be mCherry. However, even mCherry has been shown to be problematic for some specific applications [27]. In such situations, mRuby2 or TagRFP-T might prove acceptable provided sufficient controls are utilized to determine the FP is diffusing appropriately and is monomeric within the context of the fusion protein construct. The current studies demonstrate that significant progress has already been achieved in engineering monomeric red FPs, bright red FPs, and red FPs with high flickering thresholds. Combining all of these qualities into a single red FP will enable increased utilization of these remarkable proteins for single molecule and other spectroscopic studies of protein interactions.

## Figures and Tables

**Table 1:** Summary of results of FCS studies on five red FPs and mCerulean3.

	Flickering threshold (kW/cm^2^)	Optimal power (kW/cm^2^)	*D* in soln (μm^2^/s)	*D* in live cells (μm^2^/s)	D FP-BZip in cytosol (μm^2^/s)	D FP-BZip in nucleus (μm^2^/s)	Brightness published (a.u.) [ref]	*M_b_* (FPs in live cells)	*M_b_* (FP-BZip live cells)
mCherry	0.39	0.11	96.6 ± 8.1	30.0 ± 3.1	21.1 ± 8.5	8.6 ± 2.2	2.62 [6]	2.0 ± 0.2	1.65
mApple	--	0.075	122.2 ± 7.5	37.8 ± 8.5	27.4 ± 7.3	14.2 ± 4.0	6.06 [11]	5.6 ± 1.6^a^	n.d.
TagRFP-T	0.91	0.10–0.13	89.9 ± 8.0	26.6 ± 3.9	23.9 ± 4.6	7.9 ± 1.8	8.02 [13]	13.4 ± 2.5	9.8 ± 1.9
mRuby2	0.41	0.17	96.2 ± 5.6	19.1 ± 3.6	24.6 ± 4.4	9.7 ± 2.7	7.04 [9]	6.8 ± 1.6	7.0 ± 0.9
FusionRed	0.11	0.11	93.0 ± 13.7	33.2 ± 6.8	n.d.	n.d.	2.95 [8]	2.7 ± 0.6	n.d.
mCerulean3	n.d.	n.d.	92.5 ± 8.5	29.3 ± 4.4	22.6 ± 5.8	7.3 ± 2.6	n.d.	n.d.	n.d.

a Autocorrelation fitting for mApple did not provide an accurate value for *D* using a one-component fit; Therefore, data were fit using a diffusion model in which *N* was determined by including a flickering component and setting *D* equal to *D_mCherry_* (see Section 3.2 and Equation (4)).
